# Lost in translation: pluripotent stem cell-derived hematopoiesis

**DOI:** 10.15252/emmm.201505301

**Published:** 2015-07-14

**Authors:** Mania Ackermann, Steffi Liebhaber, Jan-Henning Klusmann, Nico Lachmann

**Affiliations:** 1 RG Reprogramming and Gene Therapy, REBIRTH Cluster of Excellence, Hannover Medical SchoolHannover, Germany; 2 Institute of Experimental Hematology, Hannover Medical SchoolHannover, Germany; 3 Pediatric Hematology and Oncology, Hannover Medical SchoolHannover, Germany; 4 JRG Translational Hematology of Congenital Diseases, REBIRTH Cluster of Excellence, Hannover Medical SchoolHannover, Germany

**Keywords:** granulocytes, hematopoiesis, hematopoietic stem cells, iPSC, macrophages

## Abstract

Pluripotent stem cells (PSCs) such as embryonic stem cells or induced pluripotent stem cells represent a promising cell type to gain novel insights into human biology. Understanding the differentiation process of PSCs *in vitro* may allow for the identification of cell extrinsic/intrinsic factors, driving the specification process toward all cell types of the three germ layers, which may be similar to the human *in vivo* scenario. This would not only lay the ground for an improved understanding of human embryonic development but would also contribute toward the generation of novel cell types used in cell replacement therapies. In this line, especially the developmental process of mesodermal cells toward the hematopoietic lineage is of great interest. Therefore, this review highlights recent progress in the field of hematopoietic specification of pluripotent stem cell sources. In addition, we would like to shed light on emerging factors controlling primitive and definitive hematopoietic development and to highlight recent approaches to improve the differentiation potential of PSC sources toward hematopoietic stem/progenitor cells. While the generation of fully defined hematopoietic stem cells from PSCs remains challenging *in vitro*, we here underline the instructive role of cell extrinsic factors such as cytokines for the generation of PSC-derived mature hematopoietic cells. Thus, we have comprehensively examined the role of cytokines for the derivation of mature hematopoietic cell types such as macrophages, granulocytes, megakaryocytes, erythrocytes, dendritic cells, and cells of the B- and T-cell lineage.

## Introduction

**Figure 1:**
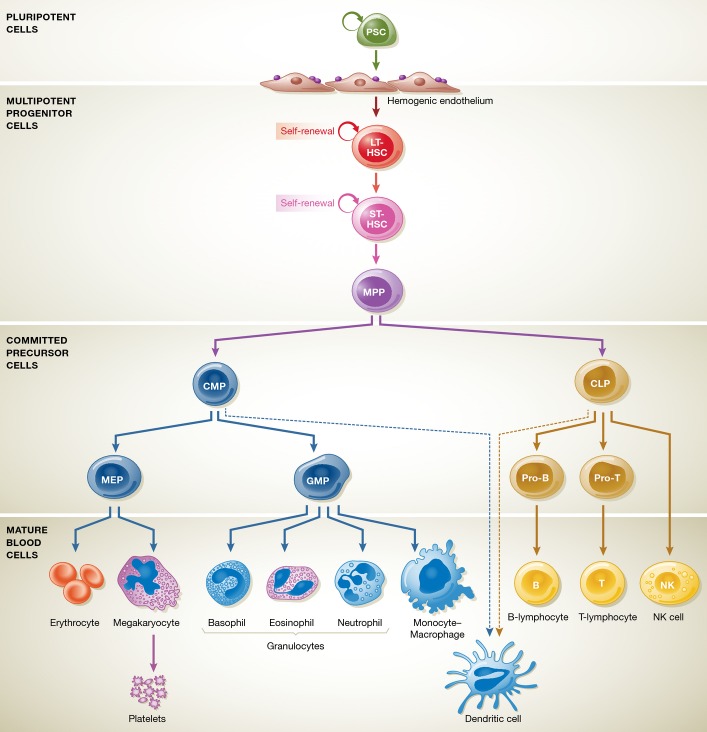
Classical scheme of murine adult hematopoietic development Multipotent LT-HSCs, with their ability for long-term reconstitution potential, can further differentiate toward ST-HSCs and also MPPs in the bone marrow. Upon subsequent differentiation, MPPs give rise to either CMPs, which have the ability to differentiate into the myeloid lineage, or CLPs, able to generate the lymphoid lineage. Following these committed progenitors, both MEPs and GMPs are able to form all differentiated cells of the myeloid lineage in the bone marrow, whereas CLPs further differentiate into pro-T cells and T cells by positive–negative selection in the thymus. Generation of B cells is ensured also by CLPs in the bone marrow following B-cell transition. Abbreviations: LT, long term; ST, short term; MPP, multipotent progenitor; HSC, hematopoietic stem cell; CMP, common myeloid progenitor; CLP, common lymphoid progenitor; MEP, megakaryocyte erythroid progenitor; GMP, granulocyte macrophage progenitor.

In 1961, James Till and Ernest McCulloch demonstrated the formation of hematopoietic colonies—comprising hematopoietic cells of multiple lineages—in the spleen of lethally irradiated mice that were transplanted with murine bone marrow (BM) cells. Based on these results, they postulated the existence of a clonogenic progenitor cell with multilineage developmental potential [colony-forming units, spleen (CFU-S)], later referred to as hematopoietic stem cell (HSC) (Till, 1961; Till & McCulloch, 1961). In subsequent work, they could demonstrate that these progenitor cells could (i) self-renew and (ii) reconstitute the entire hematopoietic system of a recipient (Becker *et al*, 1963; Siminovitch *et al*, 1963). Nowadays, hematopoiesis is understood as a hierarchal process, in which all the different specialized hematopoietic cell types are generated from a small number of definitive multipotent HSCs (see Fig1). Having these unique properties, HSC transplantation (HSCT) is applied in the clinics for the treatment of malignant and non-malignant hematopoietic disorders (Thomas *et al*, 1959; Gatti *et al*, 1968; Copelan, 2006). However, quantity and even quality of HSCs are currently the limiting factors for this therapeutic option. This is, on the one hand, due to insufficient sources for HSC isolation and inadequate storage of isolated cells. Furthermore, also immunological incompatibilities due to the multifaceted human leukocyte antigen system (known as HLA system) remain a major hurdle for HSCT (Copelan, 2006). In this regard, a new cell source, able to differentiate into all cell types of the endo-, ecto-, or mesodermal lineages, has been introduced by Shinya Yamanaka in 2006 (Takahashi & Yamanaka, 2006). These induced pluripotent stem cells (iPSCs) can be generated from different somatic cell sources by overexpression of specific transcription factors (TF) (Takahashi & Yamanaka, 2006; Yu *et al*, 2007) and may open a new chapter for the field of regenerative medicine. Although generation of iPSCs was proven for a variety of different mature cells (Takahashi *et al*, 2007; Aasen *et al*, 2008; Hanna *et al*, 2008; Kim *et al*, 2008; Haase *et al*, 2009), their proper differentiation toward transplantable therapeutic target cells of the hematopoietic lineage remains challenging. Therefore, this review aimed to highlight the recent progress within the field of hematopoietic differentiation of pluripotent stem cell sources and to address existing hurdles associated with the generation of HSCs capable for long-term reconstitution. By shedding light on the emerging factors that regulate both primitive and definitive hematopoietic development, we further provide insights into the differentiation potential of PSC sources toward hematopoietic stem/progenitor cells and mature hematopoietic cells, which may pave the way for innovative cell replacement therapies.

## Early hematopoietic development

The process of embryonic hematopoietic development is tightly regulated by the activation or repression of distinct signaling pathways. After gastrulation, cells of the epiblast ingress the primitive streak and start to differentiate toward the cells of the mesodermal lineage (Kinder *et al*, 1999). This initial formation of mesodermal cells resembles the first critical wave of hematopoietic development and is primarily regulated by the bone morphogenic protein 4 (BMP4), the fibroblast growth factor 2 (FGF2, also known as bFGF), as well as Wnt and Nodal signaling (Conlon *et al*, 1994; Flamme *et al*, 1995; Winnier *et al*, 1995; Liu *et al*, 1999). Further hematopoietic development is dependent on dorsal–ventral and anterior–posterior patterning as well as lateralization of the early mesoderm. In particular, the dorsal aorta, and hence also the emerging HSCs, develops from the ventro-posterior lateral plate (splanchnic) mesoderm, which is generated by the synergistic effects of Wnt ligands and BMPs (Beddington & Robertson, 1999; Langdon & Mullins, 2011). Also, FGF2 represents a key player during this early specification process, since it induces the up-regulation of the kinase inert domain-containing receptor (KDR, also known as vascular endothelial growth factor (VEGF) receptor 2 (VEGFR2); fetal liver kinase 1 (flk1)) on meso-dermal precursor cells. Subsequently, KDR^+^ cells can respond to VEGF, which is produced by FOXA2- and SOX7-expressing visceral endoderm (Monaghan *et al*, 1993; Takash *et al*, 2001; Kennedy *et al*, 2007; Kimura-Yoshida *et al*, 2007). This signaling interaction between cells of the mesoderm and the visceral endoderm is critical for the development of endothelial and hematopoietic cells, which is further highlighted by the lethality of a KDR knockout caused by defects in blood vessel formation (Carmeliet *et al*, 1996). In line with these observations, it has been shown that exogenous substitution of VEGF, FGF2, and transforming growth factor (TGF)-β1 (Pardanaud & Dieterlen-Lievre, 1999) could mimic the endoderm interaction that induces the hemangiopoietic potential in the associated mesoderm of chick embryos. Moreover, also signaling from the hedgehog (HH) pathway is able to substitute primitive endoderm interaction and to activate murine hematopoiesis (Dyer *et al*, 2001) by induction of a signaling cascade that includes the downstream effectors Vegf, Notch, and Runx1 (Gering & Patient, 2005).

Mesodermal cells that co-express KDR as well as Brachyury and are able to give rise to both the blood and the endothelial lineage are defined as the hemangioblast (Murray, 1932). The existence of this direct common precursor for endothelial and hematopoietic cells was already postulated for the first time in 1917 (Sabin, 2002). However, evidence of its existence was only given by an *in vitro* ESC differentiation model (Kennedy *et al*, 1997; Choi *et al*, 1998), while *in vivo* studies in mouse and zebrafish failed to conclusively confirm these findings (Myers & Krieg, 2013). Therefore, the hemangioblast rather represents a state of competence than a *bona fide* bipotential precursor cell (Amaya, 2013). During further differentiation, cells of the presumptive hemangioblast migrate to the yolk sac and contribute to the first “wave” of hematopoiesis (Ferkowicz & Yoder, 2005). This initial hematopoietic program mainly generates primitive erythroid progenitors expressing fetal hemoglobin, embryonic macrophages, and megakaryocytes. Since this phase is not able to give rise to T-lymphoid cells or even transplantable HSCs, it is defined as primitive hematopoiesis. Following this initial hemato poietic program, erythroid–myeloid progenitors (EMPs) are generated in the blood island capillaries of the yolk sac by a specialized population of endothelial cells, known as the hemogenic endothelium (HE) (Dzierzak & Speck, 2008; Lux *et al*, 2008; Yoder, 2014). Moreover, HE in the yolk sac as well as later in the para-aortic splanchnopleura can also develop into T-lymphoid progenitor cells (Yoshimoto *et al*, 2012; Boiers *et al*, 2013; Yoder, 2014). Since this phase is capable to generate “adult-like” blood cells from EMPs and lymphoid progenitors, it is defined as definitive hematopoiesis (Boiers *et al*, 2013; Yoder, 2014). However, this intermediate program does not yet generate definitive HSCs with repopulating potential.

The final step in definitive hematopoietic development is characterized by the specialization of definitive HSCs from the hemogenic endothelium that are present within the aorta-gonad-mesonephros region (AGM). Here, the proper transition from endothelial to hematopoietic cells (known as endothelial-to-hematopoietic transition (EHT)) is regulated by several specific signaling events and culminates in the formation of intra-aortic hematopoietic clusters (IAHCs) (Dzierzak & Speck, 2008). Hematopoietic transition of the HE is regulated by Notch and adenosine signaling (Gori *et al*, 2015; Jang *et al*, 2015; Jing *et al*, 2015; Lin *et al*, 2015). Although mutations in the Notch pathway lead to normal primitive hematopoiesis in the yolk sac, *runx1* expression and therefore the formation of IAHC are abolished (Burns *et al*, 2005). In this line, *Runx1* represents a crucial TF in the regulation of EHT and is highly expressed in the aortic hemogenic endothelium and IAHC (North *et al*, 2002). Once specified from the HE, HSCs leave the dorsal aorta and move toward the placenta and fetal liver for transient proliferation, after which they are finally able to colonize the bone marrow as the most important adult hematopoietic organ.

## Hematopoietic specification of pluripotent stem cells—a mirror of development

Given the importance of intrinsic and extrinsic signals for hematopoietic development *in vivo*, most *in vitro* hematopoietic differentiation protocols for PSCs try to mimic the distinct signaling cascades active during embryonic development. Similar to the importance of BMP4, Wnt, FGF2, and VEGF signaling during early embryonic hemato-poietic development, the activation of these signaling pathways has been shown to improve hematopoietic specification also upon *in vitro* differentiation of hPSCs (Winnier *et al*, 1995; Chadwick *et al*, 2003; Kennedy *et al*, 2007; Wang & Nakayama, 2009). In this respect, Kennedy *et al* (2007) demonstrated that the addition of BMP4 is essential for hemangioblast development from human PSCs. Moreover, also the cooperative effect of Wnt and BMP signaling during early hematopoietic development could be recapitulated upon *in vitro* differentiation (Wang & Nakayama, 2009).

During early stages of hematopoietic differentiation *in vitro*, PSCs give rise to cell types that express typical primitive posterior mesodermal markers, such as the apelin receptor (APLNR), platelet-derived growth factor receptor (PDGFR)α/CD140a, and KDR (Shalaby *et al*, 1997; Slukvin, 2013b; Uenishi *et al*, 2014). Moreover, differentiated cells also show the up-regulation of typical meso/endodermal TFs such as *Brachyury* (*T), MIXL1, FOXF1,* and *GATA2* (Slukvin, 2013a). Upon further differentiation, these cells acquire blast colony-forming cell (BL-CFC) potential in the presence of FGF2, similar to their *in vivo* counterparts found in the posterior region of the primitive streak, expressing KDR and T (Huber *et al*, 2004). Interestingly, overexpression of *Gata2, Lmo2, Mycn, Pitx2, Sox17,* and *Tal1* in mPSCs established and subsequently maintained a proliferative state with hemangioblast potential (Vereide *et al*, 2014). Following *in vitro* differentiation, emergence of so-called hematovascular mesodermal progenitors (HVMP) that are KDR^bright^, APLNR^+^, and PDGFRα^low/−^ has been observed from hPSCs. Moreover, HVMPs display the down-regulation of primitive streak genes and up-regulation of genes associated with angiohematopoietic development, such as *TAL1, HHEX, LMO2, GATA2, and ETV2*. At this stage, the first endothelial cells characterized by the expression of CD144 (also known as VE-cadherin) and CD31 emerge (Choi *et al*, 2012). In addition, Choi *et al* (2012) were able to identify a surface marker expression profile of CD73, CD43, and CD235a that can be used to discriminate hemogenic from non-hemogenic endothelium. In their experimental setting, only CD144^+^/CD73^−^/CD235a^−^/CD43^−^ cells were able to generate endothelial and definitive hematopoietic progenitors upon co-cultivation with OP9 stromal cells. Of note, Hirai *et al* (2003) demonstrated that the expression level of *Runx1* critically defines subpopulations within the CD144^+^ population. This finding is in line with the observation that *Runx1* is critical for the EHT during embryonic development (Chen *et al*, 2009). Following differentiation, *Sox17* regulates hemogenic endothelium (Clarke *et al*, 2013; Nakajima-Takagi *et al*, 2013) and its hematopoietic progenitors that can be further discriminated by the expression of CD43 or the pan-hematopoietic marker CD45 with a surface marker phenotype of lin^−^/CD34^+^/CD45^+^/CD38^−^ (Rafii *et al*, 2013).

## Primitive versus definitive hematopoietic development

**Figure 2:**
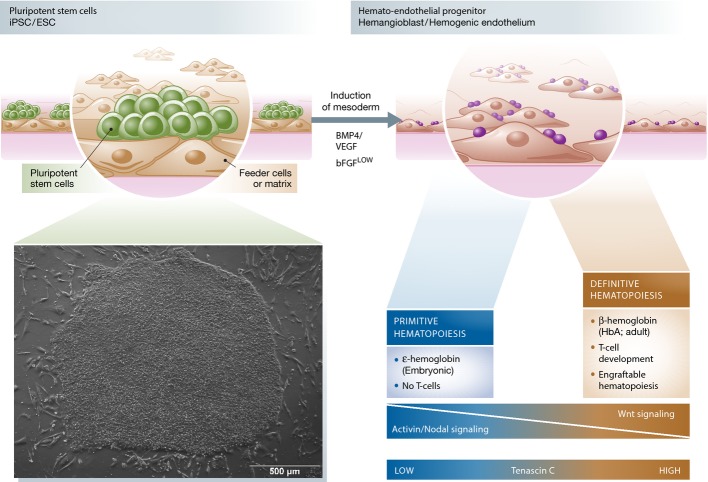
Primitive and definitive hematopoietic development *in vitro* Hematopoietic specification from pluripotent stem cells *in vitro* is primarily driven by the formation of mesodermal cells, which later gives rise to different hematopoietic cells by a hemato-endothelial progenitor. At this stage, hematopoietic differentiation *in vitro* can in principle generate cells of primitive or definitive hematopoiesis, which can be differentiated using specific experimental setups. Hematopoietic progenitor cells, which emerge during the differentiation process and are able to (i) give rise to erythroid cells that express adult hemoglobin (HbA or β-hemoglobin), (ii) give rise to T-lymphoid cells when cultured on NOTCH-delta ligand 1/4 (DL1 or DL4)-expressing OP9 cells, or (iii) multilineage reconstitute immunocompromised mice, are defined as cells derived from a definitive hematopoietic program. In contrast, hematopoietic progenitor cells that are not capable of fulfilling these criteria are defined as cells derived from primitive hematopoiesis. Although both programs can occur *in vitro*, defined signaling pathways such as Wnt, Activin/Nodal, or extracellular stimuli such as tenascin C have been proven to direct the hematopoietic program toward definitive or primitive hematopoiesis.

During mammalian embryonic development, the emergence of definitive hematopoietic “stem” cells characterized by their potential to generate B- and T-lymphoid cells as well as to repopulate an irradiated host is preceded by a wave of primitive hematopoiesis (Yoder, 2014). In this line, inducing a definitive hematopoietic program during the *in vitro* differentiation process of PSCs may resemble the prerequisite to generate HSCs with long-term engraftment potential. Probably, this switch from the primitive to definitive hematopoiesis represents the bottleneck that is hindering the efficient long-term engraftment potential of PSC-derived hematopoietic stem/progenitor cells (HSPCs) so far (Szabo *et al*, 2010; Ran *et al*, 2013) (see also Fig2).

Whereas the distinct waves of hematopoiesis are temporally and spatially separated during embryonic development *in vivo*, culture systems do not allow this clear separation, so that both developmental processes simultaneously coexist *in vitro*. It has been shown that both hematopoietic programs involve the formation of hemogenic endothelium and give rise to CD34^+^/CD45^+^ hematopoietic precursor cells. Once differentiated, primitive and definitive hematopoietic progenitor cells cannot be distinguished by differential surface marker expression but only by functional criteria, such as the generation of T lymphocytes. While in principle both programs can occur *in vitro*, the Activin/Nodal pathway triggers the development of primitive but not definitive hematopoiesis (Kennedy *et al*, 2012), leading to the hypothesis that appropriate markers can discriminate progenitors of both hematopoietic programs at very early stages of mesoderm development (see also Fig2). Indeed, a recent antibody screen from Sturgeon *et al* (2014) identified glycophorin A (CD235a) as such a marker. While KDR^+^/CD235a^+^ mesodermal cells give rise to primitive hematopoiesis, KDR^+^/CD235a^−^ cells represent precursors of a definitive hematopoietic program that are able to generate T cells upon OP9 co-cultivation. Importantly, the authors also demonstrate that the induction of a definitive hematopoietic program was driven by the Wnt–catenin signaling pathway, whereas primitive hematopoiesis was dependent on Activin/Nodal signaling (see also Fig2). Modulation of these pathways and specifically the use of Wnt agonists (CHIR99021) and Activin/Nodal antagonists led to the generation of a selected population of definitive hematopoietic progenitors (Sturgeon *et al*, 2014). Given the instructive role of Wnt signaling during early mesodermal patterning in embryonic development, the importance during *in vitro* differentiation of PSCs is not unexpected. However, it remains elusive whether its mode of action is also *in vitro* mediated by the activation of caudal-type homeobox (*Cdx*) genes, which regulate homeobox (*Hox*) expression during *in vivo* development (Ikeya & Takada, 2001; Shimizu *et al*, 2005; Pilon *et al*, 2006; Lengerke *et al*, 2008). Overall, this example clearly demonstrates the importance of understanding the physiological hematopoietic development in order to improve the *in vitro* differentiation of PSCs.

In addition to the activation of signaling cascades, TFs, or other signaling molecules, also components of the extracellular matrix (ECM) contribute to the stem cell niche and modulate *in vivo* HSC fate and development. In this respect, tenascin C (TenC) has been demonstrated to be one important component of the ECM. TenC is a highly conserved glycoprotein, mainly expressed during embryonic development and it possesses a multiplicity of binding sites for integrin cell surface receptors, proteoglycans, as well as cell adhesion molecules. In addition, it also interacts with other ECM components such as heparin, fibronectin, and collagen, suggesting complex functionality (Hsia & Schwarzbauer, 2005). In adult bone marrow, it is expressed in the endosteal regions and presumably plays a role in stress conditions (Ohta *et al*, 1998). Furthermore, TenC has been shown to improve *in vitro* maintenance of HSPCs (Zuckerman & Wicha, 1983). Although its role during embryonic development is unclear, it has recently been reported that TenC promotes hemato-endothelial development of hPSCs upon *in vitro* differentiation and—even more importantly—uniquely supports T-lymphoid commitment (Chen *et al*, 2011). The effect of extracellular factors, such as components of the ECM or blood flow, is further supported by the generation of engraftable HSCs from hiPSCs by teratoma formation (Amabile *et al*, 2013; Suzuki *et al*, 2013). Based on the necessity to provide a suitable microenvironment, developing HSCs from the teratoma were able to migrate toward the bone marrow with engraftment potential.

## Improving the generation of long-term engrafting HSPCs from hPSCs

In order to improve the poor engraftment ability of *in vitro* iPSC- or ESC-derived hematopoietic stem or progenitor cells, researchers have made big attempts to find key factors involved in HSC specification from hematopoietic mesoderm. It has been suggested that members of the *HOX* gene cluster exert an important molecular switch mediated via downstream factors such as BMP4, Activin A, or VEGF. HOX proteins are a group of highly conserved TFs, which are characterized by a DNA-binding motif termed “homeobox.” The *HOX* genes are organized into four major clusters and were shown to play important roles in embryonic organogenesis. Clusters A–C have been implicated in HSC self-renewal and regulation as well as being dysregulated in the context of several leukemia subtypes (Antonchuk *et al*, 2002; Peters *et al*, 2010). Members of the *CAUDAL* family of genes (*CDX1, CDX2,* and *CDX4*) tightly regulate *HOX* gene expression during early anterior–posterior embryonic patterning. In the zebrafish, *cdx4* is expressed in the posterior mesoderm priming hematopoietic commitment by up-regulating target *HOX* genes. Further, cdx4 initiates the generation of runx1a^+^ definitive HSCs derived from the AGM (Davidson & Zon, 2006). This implicates a strong instructive role of CDX4 during HSC specification from the hemangioblast. Indeed, the ectopic overexpression of *HoxB4* in murine HSCs leads to enhanced *ex vivo* and *in vivo* expansion, while maintaining their normal differentiation and long-term repopulation potential (Antonchuk *et al*, 2001, 2002; Kyba *et al*, 2002; Krosl *et al*, 2003; Tashiro *et al*, 2011). In normal adult hematopoiesis, HoxB4 enhances the proliferation of early cells, including HSCs, and is expressed through early stages of erythroid and granulocytic differentiation (Giampaolo *et al*, 1995; Sauvageau *et al*, 1995; Pineault *et al*, 2002). Furthermore, the overexpression of *HoxB4* and *Cdx4* in murine ESC-derived hematopoietic progenitors promotes hematopoietic mesoderm specification, increases hematopoietic progenitor formation, and enhances multilineage hematopoietic engraftment of cells in lethally irradiated adult mice (Wang *et al*, 2005b). Moreover, when iPSC-derived CD41^+^, c-Kit^+^ cells were transduced with adenoviral vectors containing *HoxB4*, the number of hematopoietic progenitor cells with colony-forming potential was significantly increased (Tashiro *et al*, 2011). In addition, early embryonic development and patterning of murine CCE-ESCs shows the activation of genes targeted by HoxB4 such as *Dll1*, *Gli2*, *Nodal*, *Sox2*, *Sim2*, *Smad2,* or *Tbx3,* whereas at later time points, HoxB4 targets genes important for specific functions, such as myeloid and lymphocyte proliferation, differentiation, and activation (Fan *et al*, 2012). Therefore, HoxB4 favors the engraftment of CCE-derived hematopoietic stem and progenitor cell in immunocompetent mice (Lesinski *et al*, 2012).

Considering the instructive role of *HoxB4* in the murine system, transient overexpression of *HOXB4* does not improve the features of human ESC- or iPSC-derived hematopoietic progenitors toward a transplantable cell population (Wang *et al*, 2005a). Unlike *HOXB4*, overexpression of the *RUNX1a* isoform improves the *in vitro* generation of hematopoietic progenitors from human ESCs and iPSCs by regulating *Brachyury*, *KDR*, *SCL*, *GATA2*, and *PU.1* (Ran *et al*, 2013). *Runx1* is expressed in the HE and important for endothelial-to-hematopoietic transition (EHT) (Chanda *et al*, 2013). In this line, ectopic expression of *RUNX1a* in human PSCs leads to hPSC-derived hematopoietic progenitors that are able for multilineage reconstitution of irradiated NOD-*scid IL2r*γ^*null*^ (NSG) mice for more than 9 weeks (Ran *et al*, 2013). Moreover, the combined over-expression of *GATA2*/*ETV2*, *GATA2*/*TAL1,* or *ER71*/*GATA2*/*SCL* can lead to the formation of endothelial cells with hemogenic potential from PSC sources (Liu *et al*, 2013; Elcheva *et al*, 2014; Shi *et al*, 2014). Alternatively, the addition of *HOXA9*, *ERG*, *RORA*, *SOX4,* and *MYB* in human PSCs favors the direct differentiation into CD34^+^/CD45^+^ progenitors with multilineage potential (Doulatov *et al*, 2013).

While defined factors such as *HOXB4*, *CDX4*, *SCL/TAL1,* or *RUNX1a* have been proven to support the hematopoietic program in murine or human PSCs, further factors have been identified to instruct terminally differentiated cells toward an immature hematopoietic phenotype. In this line, hematopoietic progenitor cells have been generated from blood or endothelial cells by the ectopic overexpression of key TFs.

**Figure 3:**
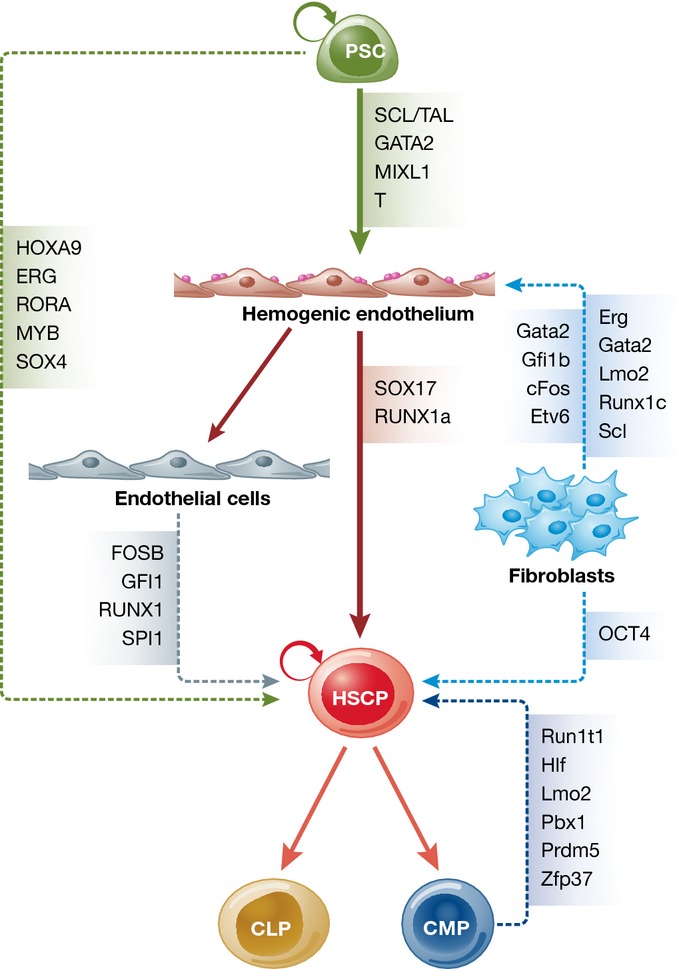
Early hematopoietic development and transprogramming strategies During hematopoietic differentiation *in vitro*, PSCs differentiate through mesodermal cells into cells of hemogenic endothelium capable to form either HSPCs or endothelial cells. This process is regulated by defined transcription factors, such as S*CL/TAL, GATA2, MIXL1, T, SOX17,* and *RUNX1a*. Further differentiation of HSPCs gives rise to CLPs or CMPs, respectively. Overexpression of *Erg, Gata2, Lmo2, Runx1c,* and *Scl* or *Gata2, Gfi1b, cFos,* or *Etv* in murine fibroblasts allows for the direct reprogramming of fibroblasts into HSPCs by a hemogenic endothelium intermediate. Alternatively, murine CMPs can be directly reprogrammed toward HSPCs by overexpression of *Run1t1, Hlf, Lmo2, Pbx1, Prdm5,* or *Zfp37*. Moreover, also endothelial cells can be directed toward HSPCs by the expression of *FOSB, GFI1, RUNX1,* or *SPI1*, whereas direct induction of HSPCs from PSCs was shown by overexpression of *HOXA9, ERG, RORA, MYB,* and *SOX4*. Abbreviations: PSC, pluripotent stem cell; HSPC, hematopoietic stem/progenitor cell; CLP, common lymphoid progenitor; CMP, common myeloid progenitor.

While the first attempt used one TF to reprogram fibroblast to immature hematopoietic precursor cells (Szabo *et al*, 2010), the combined overexpression of the transcription factors *Gata2, Gfi1b, cFos,* and *Etv6* in mouse fibroblasts efficiently induced an endothelial-like cell population (Pereira *et al*, 2013) (see Fig 3). The transient overexpression of *Run1t1, Hlf, Lmo2, Pbx1, Prdm5,* and *Zfp37* in committed myeloid and lymphoid progenitors triggered the formation of so-called induced HSCs (iHSC), which possess multilineage reconstitution potential, while being serially transplantable and showing a gene expression profile similar to *in vivo* HSCs (Riddell *et al*, 2014). Given a specific vascular niche, human endothelial cells overexpressing the TFs *FOSB, GFI1, RUNX1,* and *SPI1* are able to generate hematopoietic colonies resembling multipotent progenitor cells (MPPs), when cultured on E4EC vascular niche cells. These so-called rEC-hMPPs show colony cell-forming potential *in vitro* and faithfully engraft and reconstitute primary and secondary immune-deficient recipients (Sandler *et al*, 2014). More recently, also the ectopic expression of the TFs *Erg, Gata2, Lmo2, Runx1,* and *Scl* was proven to efficiently reprogram murine fibroblasts into blood cells through a hemogenic stage even without the need for a co-culture system (Batta *et al*, 2014) (see Fig3). Another interesting approach was introduced by Pulecio *et al*, who utilized miR-125b, a non-coding RNA, in conjunction with the TF *SOX2* to convert human fibroblasts into engraftable hematopoietic progenitor cells with mainly monocytic potential (Pulecio *et al*, 2014).

## PSC-derived hematopoiesis and the instructive role of cytokines

Pluripotent stem cells have not only been used in differentiation protocols toward hematopoietic stem and progenitor cell, but also directed toward mature blood cells. Whereas the generation of terminally differentiated hematopoietic cells has been proven for many different lineages (macrophages, monocytes, megakaryocytes, platelets, dendritic cells, T and natural killer cells as discussed below), *bona fide* HSCs—which engraft and show long-term multilineage reconstitution—have not been obtained using *in vitro* protocols so far.

The induction of specific signaling pathways by the addition of key cytokines and/or co-cultivation with instructive feeder cells is substantial for the generation of hematopoietic progenitors and mature blood cells from human PSC sources, which are usually cultured under serum-free conditions. In normal adult hematopoiesis *in vivo*, the specification of mature cell types from multipotent HSCs is positively regulated by instructive cytokine signals, that is by the activation of crucial TFs (Zhu & Emerson, 2002). It is still under debate, whether cytokines have a rather selective than a lineage-instructive function during the commitment of multipotent hematopoietic progenitors (reviewed in Endele *et al*, 2014). However, TPO and erythropoietin (EPO) were shown to support erythro-megakaryocytic lineage commitment of a common TER119^+^/4A5^+^ precursor (Vannucchi *et al*, 2000), while macrophage colony-stimulating factor (M-CSF) and granulocyte macrophage colony-stimulating factor (GM-CSF) signals drive the differentiation toward monocytes/granulocytes from multipotent progenitors (Kondo *et al*, 2000; Pawlak *et al*, 2000; Niwa *et al*, 2011). It seems reasonable that any blood cell derived from PSC has to pass through a transient progenitor cell state. Protocols for the expansion of HSPCs have therefore been adapted for the derivation of *in vitro* HSPCs from PSCs. Cytokines, such as SCF, TPO, FLT3-L, IL-6, and aryl hydrocarbon receptor signaling antagonizers (i.e., the small molecule StemRegenin), drive the expansion of cord blood CD34^+^ HSPCs while maintaining their CFU potential over a period of 10 days in culture (Haemmerle *et al*, 2014). The addition of IL-3, however, favors the expansion and differentiation of CD34^+^ HSPCs on the expense of engraftment and reconstitution *in vivo* (Du *et al*, 2014). Similarly, these cytokines have been utilized to support the generation of HSPCs from PSCs *in vitro* (Uenishi *et al*, 2014).

For early differentiation of hiPSCs and hESCs, most protocols rely on the addition of SCF, BMP4, or VEGF for the induction of mesodermal differentiation or the spontaneous differentiation within embryoid bodies (EBs) (Olivier *et al*, 2006; Pick *et al*, 2007; Yokoyama *et al*, 2009; Niwa *et al*, 2011; Ferrell *et al*, 2015; Toscano *et al*, 2015). Generated EBs can be subsequently either plated in co-culture systems with, for example, OP9, AFT024, or C3H10T1/2, or further differentiated with the help of special media, such as APEL, BPEL, or StemSpan (Chang *et al*, 2006; Olivier *et al*, 2006; Pick *et al*, 2013; Ferrell *et al*, 2015; Vanhee *et al*, 2015) (summarized in Table1).

**Table 1:** Generation of mature hematopoietic cells from pluripotent stem cell sources.

	Cell type	PSC source	Embryoid bodies	FCS	Feeder cells	Cytokines	Reference
Myeloid cells	Erythrocytes	hESC (H1)	Yes	Yes	No	bFGF, VEGF, EPO, SCF, nFlt3-L, IL-3, IL-6, G-CSF, TPO	Chang *et al* (2006)
		hESC (H1)	No	Yes	S17 and FH-B-hTERT (mFL stroma cells)	Clonogenic assay: SCF, GM-CSF, IL-3, EPO	Qiu *et al* (2005)
		hESC	No	Yes	mFL stroma cells	Clonogenic assay: SCF, IL-3, IL-6, TPO, G-CSF, EPO	Ma *et al* (2008)
		hESC (H1), hiPSC	No	Yes	OP9, MS5	TPO, IL-3, IL-6, Flt3-L, SCF, EPO	Dias *et al* (2011)
		hESC (H1)	No	Yes	FH-B-hTERT, MS5	IL-3, BMP4, Flt3-L, SCF, EPO, IGF-1	Olivier *et al* (2006)
	Megakaryocytes/platelets	hESC (HES3, Envy, MEL1)	Yes	No	No	BMP4, VEGF, bFGF, SCF, TPO, IL-3	Pick *et al* (2013)
		hESC (WA01)	Yes	Yes	OP9	BMP4, VEGF, IL-3, Flt3-L, TPO, SCF, EPO	Vanhee *et al* (2015)
		hESC (MA09, NED07), hiPSC	No	No	No	BMP4, VEGF, bFGF, TPO, SCF, Flt3-L, IL-3, IL-6, IL-9	Feng *et al* (2014)
		hESC (HuES3, MA01, MA09)	No	No	OP9, C3H	BMP4, VEGF, IL-6, IL-9, IL-11, bFGF, TPO, SCF	Lu *et al* (2011)
	Granulocytes	hESC (KhES-3)	Yes	Yes	No	IGF-II, VEGF, SCF, Flt3-L, TPO, G-CSF	Saeki *et al* (2009)
		hESC (KhES-3)	Yes	Yes	OP9	BMP4, SCF, Flt3-L, IL-6, TPO, G-CSF	Yokoyama *et al* (2009)
		hESC (KhES-1, 3), hiPSC	Yes	No	No	BMP4, VEGF, SCF, TPO, Flt3-L, IL-3, G-CSF	Niwa *et al* (2011)
		hiPSC	Yes	No	No	IL-3, G-CSF or GM-CSF	Lachmann *et al* (2015)
		hESC (H1, H9), hiPSC	No	Yes	OP9	GM-CSF, G-CSF, IL-3, IL-5	Choi *et al* (2009)
	MΦ	hESC (HUES-2, KCL001, 002)	Yes	No	No	IL-3, M-CSF	Karlsson *et al* (2008), van Wilgenburg *et al* (2013)
		hESC (H9), hiPSC	Yes	No	No	IL-3, M-CSF, or GM-CSF	Lachmann *et al* (2015)
		hESC (H1, H9), hiPSC	No	Yes	OP9	GM-CSF, M-CSF, IL-1b	Choi *et al* (2009)
	DCs	hESC (H9)	Yes	hAS	No	SCF, Flt3-L, GM-CSF, IL-3, TPO, IL-4, TNF-α	Su *et al* (2008)
		hIPSC	No	Yes	OP9	GM-CSF, M-SCF, IL-4, TNF-α	Senju *et al* (2011)
		hESC (H1, H9), hiPSC	No	Yes	OP9	GM-CSF, IL-4, TNF-α	Choi *et al* (2009)
Lymphoid cells	NK cells	hESSC (H1, HES-2), hiPSC	Yes	Yes	OP9-DL4	BMP4, bFGF, Activin A, VEGF, IGF-1, IL-6, IL-11, SCF, IL-3, EPO, TPO, IL-13, Flt3-L, IL-15	Sturgeon *et al* (2014)
		hESC (H9), hiPSC	Yes	hAS	OP9-DL1	BMP-4, VEGF, SCF, IL-3, Il-6, TPO, EPO, IL-7, Flt3-L, IL-15	Ferrell *et al* (2015)
		hESC (H9)	No	Yes	S17, AFT024 (mFL cells)	IL-15, IL-3, IL-7, SCF, Flt3-L	Woll *et al* (2005)
	T cells	hESC (H1)	Yes	Yes	OP9-DL4	BMP-4, bFGF, Activin A, VEGF, IL-6, IGF-1, IL-11, SCF, EPO, TPO, Flt3-L, IL-7, IL-15	Kennedy *et al* (2012)
		hESSC (H1, HES-2), hiPSC	Yes	Yes	OP9-DL4	BMP4, bFGF, Activin A, VEGF, IGF-1, IL-6, IL-11, SCF, IL-3, EPO, TPO, IL-3, Flt3-L, IL-7	Sturgeon *et al* (2014)
		hESC (H1, H9), hiPSC	No	Yes	OP9-DL1, OP9-DL4	BMP-4, bFGF, VEGF, TPO, SCF, IL-6, IL-3, IL-7, Flt3-L	Uenishi *et al* (2014)
		hESC (H1)	No	Yes	OP9, OP9-DL1	Flt3-L, IL-7, SCF	Timmermans *et al* (2009)
	B cells	hESC (H1, H9, ES03),	Yes	Yes	OP9	BMP4, VEGF, FGF1, bFGF, SCF, Flt3-L, TPO, GM-CSF, IL-2, IL-4, IL-15, G-CSF, IL-3, IL-6, IL-7	Zambidis *et al* (2008)
		hIPSC	No	Yes	OP9, MS5	IL-7, IL-3, SCF, Flt3-L	French *et al* (2015)

PSC, pluripotent stem cells; hESC, human embryonic stem cells; hiPS, human-induced pluripotent stem cells; bFGF, basic fibroblast growth factor; VEGF, vascular endothelial growth factor; EPO, erythropoeitin; SCF, stem cell factor; Flt3-L, FMS-like tyrosine kinase 3 ligand; IL-3, interleukin-3; IL-6, interleukin-6; G-CSF, granulocyte colony-stimulating factor; TPO, thrombopoietin; FL, fetal liver; GM-CSF, granulocyte–macrophage colony-stimulating factor; BMP4, bone morphogenic protein 4; IGF-1, insulin-like growth factor 1; IL-9, interleukin-9; IL-11, interleukin-11; IGF-II, insulin like-growth factor II; M-CSF, macrophage colony-stimulating factor; IL-1b, interleukin-1b; IL-4, interleukin-4; TNF-α, tumor necrosis factor-alpha; IL-7, interleukin-7; FGF1, fibroblast growth factor 1; IL-15, interleukin-15; hAS, human antibody serum; MΦ, macrophages; DCs, dendritic cells; NK, natural killer cells.

Different studies have shown the production of erythrocytes from hiPSCs or hESCs using VEGF, SCF, BMP4, Flt3-L, IL-3, IL-6, and EPO, while some protocols additionally used TPO, hydrocortisone, or insulin-like growth factor 1 (IGF-1) (Qiu *et al*, 2005; Chang *et al*, 2006; Olivier *et al*, 2006; Ma *et al*, 2008; Dias *et al*, 2011). Although the generation of such cells has been proven, successful transfusion of mature red blood cells (RBC) is still hampered primarily due to the low efficiency of the differentiation process. For clinical application, one unit should at least contain 10^10^ *in vitro* derived cells, whereas 10^12^ RBCs are desirable, a cell number that is not reached by far with the current protocols from PSCs (Dorn *et al*, 2015). Likewise, the resulting erythrocytes were mostly nucleated and contained only embryonic or fetal, but not adult hemoglobin, favoring the concept of primitive hematopoiesis. Alternatives, such as immortalized iPSC-derived erythroblast (imERYPCs), are highly promising and maybe used as a safe and constant supply for RBC transfusion (Hirose *et al*, 2013).

Similarly, TPO, SCF, or IL-3 also seem to be essential for the development of megakaryocytes and platelets from peripheral blood-derived CD34^+^ cells as well as hESCs/hiPSCs (Pick *et al*, 2013; Nakamura *et al*, 2014; Vanhee *et al*, 2015). Moreover, also interleukin-9 (IL-9) and interleukin-11 (IL-11) were reported to improve the quality of the produced platelets (Lu *et al*, 2011). Most recently, a feeder- and serum-free protocol with collagen IV was established, which yields highly pure CD41a^+^/CD42b^+^ double-positive mature megakaryocyte and platelet populations from hiPSCs and hESCs (Feng *et al*, 2014). Human iPSC-derived platelets can be generated as HLA-ABC negative, can be frozen and share functional features with peripheral blood-derived platelets both *in vitro* and *in vivo* (Feng *et al*, 2014; Nakamura *et al*, 2014). As the efficient dose for a transfusion would be approximately 300–600 × 10^9^ platelets, yields of 6 platelets per megakaryocyte progenitor from iPSC cultures are clearly too low for a clinical translation (mature MKs produce 2,000–10,000 platelets) (Feng *et al*, 2014). However, it might be possible to further improve the current protocols by altering sheer stress and matrix interactions in combination with GMP-compliant media or expandable iPSC-derived megakaryocytic cell lines (Moreau *et al*, 2013; Nakamura *et al*, 2014).

Peripheral blood-derived CD34^+^ cells can be efficiently differentiated into neutrophil or eosinophil granulocytes by the use of granulocyte colony-stimulating factor (G-CSF) and IL-6 or the combination of IL-3 and IL-5 on OP9 feeder cells, respectively (Choi *et al*, 2009). A similar protocol was used to generate neutrophils and eosinophils from human ESCs and iPSCs via a GM-CSF-expanded intermediate CD235a^−^/CD41a^−^/CD34^+^/CD45^+^ cell type. The resulting populations were essentially pure and similar to peripheral blood-derived granulocytes regarding marker expression, function, and morphology. Another approach using an EB-based, feeder-free protocol with the addition of insulin-like growth factor II (IGF-II), VEGF, SCF, Flt3-L, TPO, and G-CSF could only generate neutrophils from hESCs with lower efficiencies (Saeki *et al*, 2009). Recently, the combination of SCF, TPO, IL-3, or G-CSF was shown by several groups to faithfully induce mature neutrophils from human pluripotent cell sources (Yokoyama *et al*, 2009; Niwa *et al*, 2011; Lachmann *et al*, 2015). Limitations to most systems comprise the low yields, especially in feeder-free conditions, the dependence on expensive cytokine cocktails, or the selection of a suitable ESC/iPSC line that efficiently differentiates into myeloid cells. Although PSC-derived granulocytes show similarities to their *in vivo* counterparts, discrepancies in the formation of neutrophil extracellular traps (NETs) or migration toward hIL-8 were reported (Lachmann *et al*, 2015), arguing for cells in different maturation stages within granulocytic differentiation. IPSC-derived granulocytes hold great potential for the treatment of infections and septicemia in neutropenic patients. However, it is still unclear whether the *in vitro* derived granulocytes are fully functional after transfusion. In addition, suitable cell numbers for individual granulocyte transfusions and storage conditions remain critical obstacles that have not been addressed to date. In particular, the latter is of interest, as granulocytes are known to have a short life span after leukapheresis. Since granulocytes are generated at 37°C using cell culture media, it remains elusive whether PSC-derived granulocytes can be stored in aliquots ready for on-demand therapeutical use.

Since macrophages and granulocytes descend from a common progenitor (i.e., GMP), their early differentiation steps from HSCs or PSCs toward the myeloid lineage are principally guided by the same molecular cues. In order to obtain monocytes and macrophages *in vitro*, most protocols use a combination of SCF, IL-3, M-CSF, or GM-CSF (Karlsson *et al*, 2008; Choi *et al*, 2009; van Wilgenburg *et al*, 2013; Lachmann *et al*, 2015) (see also Table1). *In vitro* derived macrophages show high functional and morphological similarities to PB-derived counterparts, but again their *in vivo* function upon transfusion has to be evaluated using suitable animal models. Here, even smaller numbers of monocytes or macrophages might be sufficient for a clinical benefit as a bridging therapy for infectious diseases or as tissue resident macrophages in organotropic transplantation scenarios (Happle *et al*, 2014; Lachmann *et al*, 2014; Suzuki *et al*, 2014a,b).

Dendritic cells (DCs) are antigen-presenting cells, which can be derived from both myeloid and lymphoid developmental pathways. DCs have been differentiated from bone marrow or peripheral blood CD34^+^ cells using GM-CSF, Flt3-L, SCF, IL-4, and tumor necrosis factor-α (TNF-α) (Lutz *et al*, 1999; Choi *et al*, 2009). Similarly, hESC/hiPCS-derived DCs can be generated with the help of mainly GM-CSF, IL-4, or TNF-α through a transient myeloid intermediate (Su *et al*, 2008; Choi *et al*, 2009; Senju *et al*, 2011) (see also Table1).

The *in vitro* differentiation of hESCs/hiPSCs into cells of the lymphoid lineage is strictly dependent on the co-culture with stromal cells, such as OP9, AFT024, or MS-5, and much lower in efficiency compared to the *in vitro* generation of myeloid or erythro-megakaryocytic cells. In this line, several protocols have been established to generate mature natural killer cells (NK) from human iPSCs or ESCs, commonly using the hematopoietic cytokines SCF, Flt3-L, IL-3, IL-7, and IL-15 (Woll *et al*, 2005; Sturgeon *et al*, 2014; Ferrell *et al*, 2015) (see also Table1).

As Notch signaling was shown to be essential for T lymphopoiesis (Mohtashami *et al*, 2010), derivation of T cells from hiPSCs/hESCs was achieved with the help of OP9 stromal cells constantly overexpressing the Notch ligand Delta-like 1 or 4 (DL1 or DL4, respectively). A combination of different cytokines was shown to faithfully induce T-cell differentiation using either thymopentin, OP9 co-culture, or transplantation-based differentiation protocols starting from *in vivo* progenitor populations or ESCs (Timmermans *et al*, 2009; Kennedy *et al*, 2012; Sturgeon *et al*, 2014; Uenishi *et al*, 2014; Zhu *et al*, 2015) (see also Table1). Early protocols for the generation of *in vitro* B cells used OP9 stromal cells and a combination of Flt3-L and IL-7 (Cho *et al*, 1999; Zambidis *et al*, 2008). When cultured on MS-5 stroma in the presence of IL-7, SCF, Flt3-L, and IL-3, hiPSC-derived CD34^+^ cells differentiated into CD19^+^CD10^+^ B cells that undergo *in vitro* VDJ recombination and express cell surface IgM (French *et al*, 2015).

Taken together, current protocols for the *in vitro* generation of mature blood cells from human pluripotent cells demonstrate the importance of the key cytokines SCF, IL-3, and IL-6 for myeloid differentiation, whereas SCF, TPO, and EPO seem to be instructive for a rather erytho-megakaryocytic lineage decision. In contrast, the generation of lymphoid progenitors from PSCs involves signaling by SCF, Flt3-L, IL-3, IL-7, and/or IL-15 and additionally relies on co-culture systems with OP9 cells. This dependency might reflect the fact that the current protocols for the derivation of hematopoietic cells from PSCs are not yet able to mimic the complex cues needed for the induction of a definitive hematopoietic program. Further improvement of environmental and intrinsic signaling pathways could lead to an enhanced, large-scale production of fully functional PSC-derived blood cells.

While large-scale generation of suitable iPSC-derived cells under GMP-compliant conditions remains the next hurdle for the successful transfer toward the clinics, also the functionality of iPSC-derived cells in suitable *in vivo* mouse models remains elusive. Different mouse models favoring the engraftment of human hematopoietic cells have been developed, either expressing mutants of the Kit receptor (Cosgun *et al*, 2014) or different human cytokines such as CSF1 alone (Rathinam *et al*, 2011) or in combination with IL3, CSF2, and THPO (Rongvaux *et al*, 2014). While current protocols have shown the functionality of mature cells mostly *in vitro*, transfer of cells into the aforementioned humanized mouse models is still hampered by the rather low output of differentiated cells from PSC sources. Investigating new ways of differentiation to either increase the yield or quality of cells may pave the way for innovative cell replacement strategies using iPSC-derived mature hematopoietic cells.

## Conclusion

A multitude of differentiation protocols has proven the efficient generation of mature hematopoietic cells from PSC sources. However, the generation of lymphoid cells or erythrocytes still remains challenging and highly inefficient. Moreover, the directed differentiation of PSCs into HSPC, with the ability to efficiently reconstitute xenograft models long term, is still hampered. This indicates that most protocols direct the differentiation process toward primitive hematopoietic development. Here, a better understanding of defined factors regulating both the primitive and the definitive hematopoietic development *in vivo* might also help to further fine-tune the *in vitro* differentiation process. Considering the hematopoietic differentiation from PSC as a finely orchestrated and dynamic process, the use of defined cytokines only may skew or bias the *in vitro* hematopoietic differentiation toward cell types not applicable for clinical use. Here, cell–cell interactions, the extracellular matrix (ECM), or cytokines from cell niches acting either in an autocrine or paracrine fashion during the *in vitro* differentiation process are rarely considered yet. As an example, Sturgeon *et al* (2014) elegantly demonstrated that modulation of the Wnt pathway during hematopoietic specification *in vitro* can lead to the generation of definitive hematopoiesis, highlighting the importance of an improved understanding. Further understanding of the finely tuned influence from niche and stromal cells on hematopoietic progenitors (i.e., via up-regulation of TF) will be essential for the improved, large-scale production of PSC-derived blood cells *in vitro*.

**Pending issues**

What factors (intrinsic and extrinsic) are regulating primitive versus definitive hematopoietic development?

Will the identification of those factors lead to improved differentiation of PSCs into long-term engrafting HSPCs?

Do we bias *in vitro* hematopoietic differentiation, resulting in hematopoietic cells not similar to their *in vivo* counterparts?

How can we improve protocols for the generation of functional lymphoid cells and red blood cells from PSCs?

Do suitable xenograft models that are able to support multilineage engraftment of human iPSC-derived hematopoiesis help us to understand the human hematopoietic differentiation?

Where can we apply PSC-derived mature hematopoietic cells for innovative treatment options and how should we proceed?

What are the obstacles associated with up-scaling of GMP-compliant hematopoietic differentiation protocols for regenerative therapies?

## For more information

For more information on pluripotent stem cells and multipotent stem cells please visit:

Stem Cell Information, National Institute of Health (NIH)

http://stemcells.nih.gov/info/basics/Pages/Default.aspx

International Society for Stem Cell Research

http://www.isscr.org

PSC-derived hematopoietic cells and their use for rare diseases

http://www.rebirth-hannover.de/en/research/research-groups/area-b1/unit-68.html

## Abbreviations

Engraftment: Incorporation of donor cells, e.g. HSC, into a new host, thereby giving rise to donor-derived hematopoiesis.
Epiblast: During early embryonic development, the inner cell mass (ICM) of the blastocyst segregates into the bilaminar disc, which comprises the epiblast and the primitive endoderm. The epiblast (primitive ectoderm) further undergoes gastrulation and is able to form the three germ layers (endoderm, ectoderm and mesoderm) and some extra-embryonic components.
Hematopoietic stem cell (HSC): The concept of blood formation is a hierarchical process referred to as hematopoiesis that is based on the multi-potent hematopoietic stem cell (HSC), which is able to reconstitute the entire hematopoietic system.
Hemogenic endothelium: A specialized population of endothelial cells present in the developing embryo able to give rise to hematopoietic cells.
Induced pluripotent stem cells (iPSC): A pluripotent cell-type with the capacity to differentiate towards all cells of the three germ-layers (ectoderm, mesoderm, endoderm) and induced by reprogramming of a somatic cell by defined factors.
Mesoderm: One of the three germ layers formed upon gastrulation during early embryonic development. Mesodermal progenitors give rise to e.g. cartilage, muscles, connective tissues, blood, heart and kidney.
Transdifferentiation: Describes the direct conversion of a somatic cell-type into another somatic cell, without going through an intermediate pluripotent status. This process can occur naturally during development or be induced by overexpression of lineage-specific transcription factors
